# ‘You truly are the worst kind of racist!’: Argumentation and polarization in online discussions around gender and radical‐right populism

**DOI:** 10.1111/bjso.12557

**Published:** 2022-06-29

**Authors:** Katarina Pettersson, Inari Sakki

**Affiliations:** ^1^ University of Helsinki Helsinki Finland; ^2^ University of Eastern Finland Kuopio Finland

**Keywords:** critical discursive psychology, female politicians, online discussions, political polarization, radical‐right populism

## Abstract

The role of women in populist and radical right‐wing parties is a topic that has gained increased scholarly attention. The aim of this article is to add to this literature by analysing how a female right‐wing populist leader becomes positioned in online interactions in the hybrid media system. In doing so, the study seeks to make a twofold contribution to research on populist and radical right discourse online: to explore the ways in which notions related to gender and radical‐right populism are constructed in such discourse and to shed light on their argumentative character. The study applies a critical discursive psychological approach to study these discursive patterns in two interrelated social media datasets, comprising discussion threads from Facebook and Twitter. The study shows how, through argumentation and dialogue, the commenters’ position both each other and the female populist leader as fit or unfit, racist or non‐racist, patriotic or non‐patriotic and as a victim or culprit of hate‐speech and misogyny. The implications of these findings for social psychological research on radical‐right populism, political polarization and online hate‐speech are discussed.

## INTRODUCTION

Riikka Purra was chosen as the first female president of the populist radical right Finns Party (Fin: *Perussuomalaiset*) on August 14th, 2021. Her predecessor Jussi Halla‐aho, twice prosecuted for hate‐speech against minorities and internationally known as one of the most influential actors within the Finnish far‐right and anti‐immigration circles, had shifted the party's ideology and agenda away from its agrarian roots and towards the far‐right and nationalist direction (cf. Saresma, Karkulehto & Varis, 2021). In public debates, Purra was described as ideologically adjacent to Halla‐aho, a ‘hard politician’ with equally strong nationalist and anti‐immigration stances, yet with a ‘softer’, less polemic rhetorical profile (Waris, 2021). Indeed, despite her outspoken nationalist agenda, Purra is in many ways an atypical populist leader: she is a highly educated, upper middle class urban politician in her forties, she has for years been blogging actively about her vegetarian diet, and finally, she is a woman.

As the examples of Marine Le Pen in France, Frauke Petry in Germany, Siv Jensen in Norway and Pia Kjaersgaard in Denmark demonstrate, despite the parties remaining male‐dominated, women have achieved prominent positions in populist radical right parties during the last decade (Meret, Siim & Pingaud, 2016). Upon her election, Riikka Purra's somewhat contradictory profile rendered her the target of public and media scrutiny in Finland and beyond. Representing the biggest opposition party in the Finnish national parliament, she quickly became positioned as a counterforce to the Finnish female‐dominated government led by Prime Minister Sanna Marin. In October 2021, *Suomen Kuvalehti (SK)*, one of the biggest news magazines in Finland, published an extensive interview with Purra. The article discussed her path to become a successful politician, from childhood and the early death of her mother, her time as a diligent and somewhat introverted student of political science, her traumatic experiences of romantic relationships, to her present ideological position and claim that ‘there is no room for empathy in politics’ (Suonpää, 2021). Following the publication of the article, Purra tweeted about it, linking to the Facebook page of the magazine and quoting a derogatory comment directed at her:

“The cold shivering disgusts as this woman opens her mouth, the shivering disgust you feel towards bloodsuckers like horseflies, ticks.”

You can find a lot of such comments on the SK fb page under the story about me.

Please read the story for yourself. Link

Riikka Purra, tweet, October 8th, 2021: https://twitter.com/ir_rkp/status/1446686567821611009

Purra's tweet touches upon the topical and contested issue of hate‐speech in political and societal debates. Purra's tweet highlights the derogatory online speech that was directed at her as a political figure, elicited by the interview with her in a nationwide magazine. The present study approaches this topic by exploring how public perceptions and negotiations of gender and populist politics are constructed in argumentation, contestation and polarization in an everyday talk in the online sphere. More precisely, we deploy a critical discursive psychological perspective to analyse discussions about Purra in the hybrid media environment (Chadwick, 2017), that is, in the connected discussions on Facebook and Twitter about the SK‐article about her. Before introducing the aims of the study in more detail, we discuss, first, existing literature on public and online debates on female (populist) politicians and, second, social psychological approaches to studying the discursive patterns of such debates.

## FEMALE (POPULIST) POLITICAL LEADERS

A substantial amount of research has investigated the ‘double bind’ that female political leaders struggle with: on one side, whereas stereotypically masculine traits such as ambition and power are regarded as essential characteristics of a political leader, women who display ‘too much’ of such features become targets of public accusations of trying to conceal their femininity (Hall & Donaghue, 2012; Sorrentino & Augoustinos, 2016). On the other side, however, female politicians coming across as ‘too feminine’ can rapidly lead to criticism of ‘playing the gender card’ and of utilizing their womanhood for strategic purposes (Sakki & Martikainen, 2022).

The double bind of the identity management of female political leaders – that between hard, masculine traits on one side, and soft, feminine ones on the other – can be regarded as particularly delicate when it comes to female leaders in populist and radical right movements. This is because apart from their general strong hostility towards and harsh rhetoric regarding multiculturalism, immigration and asylum‐seeking (e.g., Sakki & Pettersson, 2016), these parties also tend to take pronounced anti‐gender positions, resisting the rights of sexual minorities and structural measures to promote gender equality (Norocel, Saresma, Lähdesmäki & Ruotsalainen, 2020; Pettersson, 2017; Ylä‐Anttila & Luhtakallio, 2017). Research examining the rhetoric of the French leader of the far‐right *Rassemblement National*, Marine Le Pen, and the former president of the Danish People's Party, Pia Kjaersgaard, in Denmark (Meret, 2015; Meret et al., 2016) has shed light on how these politicians negotiate their positions as women, on one side, and as representatives of parties promoting patriarchal ideologies, on the other, for instance, through interlacing their positions as mothers and national leaders in their talk.

Studies, for instance, in the U.S. (e.g., Carlin & Winfrey, 2009), Australia (Hall & Donaghue, 2012) and the Nordics (Sakki & Martikainen, 2022) have shown that in comparison to their male colleagues, female political leaders of all political orientations face considerable public scrutiny, criticism and even hate‐speech. As argued, for instance, by Tileagă (2019), the era of digitalisation has provided a fruitful ground for misogyny, as it can easily be produced, circulated and spread online, with consequences for the public perception of women (politicians) at different levels of society. As demonstrated by Sakki and Martikainen (2022) in their study of online misogynist discourse about the Finnish PM Sanna Marin, online discrimination against female political leaders can take many different shapes, ‘from milder forms of derogation, scorn and other‐condemning practices to harsher forms of belittlement, humiliation and animalistic dehumanisation’ (Sakki & Martikainen, 2022, abstract). Sakki and Martikainen conclude, however, that online constructions of hatred, dehumanization and misogyny have the potential to evoke voices of opposition and resistance; thus, they call for researchers to explore this avenue. The present study is an attempt to do so, that is, to investigate the dialogical construction of positions and counter‐positions for a female populist leader in online interactions.

## THE ONLINE SPACE AS A SITE FOR HATE‐SPEECH

Online platforms and social media, such as Facebook and Twitter, have become important sites for political struggle (Persson, 2017) and mobilization (Chadwick & Stromer‐Galley, 2016; Nelimarkka, Laaksonen, Tuokko & Valkonen, 2020). It is a sphere of the social world where a significant part of people's everyday claims‐making about issues such as (in)equality, immigration, multiculturalism and asylum‐seeking takes place (Burke & Goodman, 2012; Goodman & Burke, 2010; Merrill & Åkerlund, 2018). Indeed, the growing importance of social media for both political and everyday discursive meaning‐making is significantly influencing not only the media landscape but also transforming cultures and societal‐level discourses (Törnberg & Uitermark, 2021). Recent research suggests that social media platforms should not be regarded as comprising separate ‘echo chambers’ or ‘filter bubbles’, where ideologically like‐minded people interact only amongst each other, but rather, as sites where considerable exchange between conflicting views and identities takes place, which foments societal polarization (Törnberg, Andersson, Lindgren & Banisch, 2021).

Due to its growing importance in our digital era, discursive researchers have begun to show increasing interest in studying the online sphere as a space for social interaction and constructions of meaning, for instance, when it comes to talking about issues related to ‘race’. As Billig, (2001) has shown, and as a plethora of subsequent studies has confirmed, talk about race‐related matters tends to be formulated in more extreme ways in the online as compared to physical interactions (Bilewicz & Soral, 2020; Burke & Goodman, 2012; Cervone, Augoustinos & Maass, 2021; Merrill & Åkerlund, 2018; Pettersson & Augoustinos, 2021). Research indicates that social media platforms such as Facebook and Twitter have become fertile breeding grounds for the production of racist, Islamophobic and anti‐Semitic discourses (e.g., Ben‐David & Matamoros‐Fernández, 2016; Burke et al., 2020; Burke & Goodman, 2012). Similarly, the online context has become an environment where misogyny – a culturally bound system that promotes men's sense of superiority and hostile and derogatory treatment of women (e.g., Saresma et al., 2021; Tileagă, 2019) – is produced and spread, not least when it comes to hatred directed at women in powerful and public positions, such as politics (cf. Sakki & Martikainen, 2022; Tileagă, 2019). Indeed, despite efforts to monitor and control hateful online content (cf. Merrill & Åkerlund, 2018), for politicians and lay people alike, the Internet provides tools for expressing derogation and hate in indirect, non‐verbal ways (e.g., through images and links to external sources) on one hand, and anonymously, on the other hand, which both may account for the online language being more extreme than in face‐to‐face interactions.

In their overview of the functions, consequences, and reappropriation of derogatory and hateful language both onsite and online, Cervone et al. (2021) approach hate speech as ‘an extreme form of derogatory language, which […]involves the expression of hate and/or the encouragement of violence against others based on their real or assumed membership in a given category’ (Cervone et al., 2021, p. 81). Yet, what counts as hate‐speech remains a contested issue, and difficulties arise in particular when the task is to determine whether a particular utterance, related for instance to political matters, falls into the realm of free or hate‐speech (e.g., Pettersson, 2019).

An interesting and for our present purposes particularly relevant approach to hate‐speech is offered by David Boromisza‐Habashi, who approaches the concept from a cultural and ethnographic perspective. Boromisza‐Habashi (2012) conceptualizes hate‐speech as a *cultural practice* that is rooted in its surrounding historical, economic and political contexts, attached to a multitude of (conflictual) meanings and interpretations, and competing moral systems, including norms and cultural beliefs. Rather than taking a top‐down approach, determining the meaning of hate‐speech and examining manifestations thereof in society, Boromisza‐Habashi proposes to explore the topic from a diverse direction, that is, to study the public use and local meanings attached to the term. Inspired by this approach, in the present study we are not interested in any ‘de facto’ borders between hate‐speech and free speech, but rather, to examine the ways in which the term is deployed in online interactions to serve specific social and political functions.

## ARGUMENTS, COUNTER‐ARGUMENTS AND SEMANTIC BARRIERS IN THE ONLINE SPACE

Billig's (1985, 1987) classic work in rhetorical social psychology posited that individuals' thinking, reasoning and attempts to make sense of the (social) world are matters of continuous argumentation and counter‐argumentation between alternatives. Such argumentation, according to Billig, takes place both within and between individuals. In social interaction – be it onsite or online – therefore, any claim is always presented in relation to anticipated or present counterclaims. When it comes to debates over political and ideological issues, such as constructions and accusations of racism, these debates must therefore always be examined in their argumentative context, that is, as part and parcel of the social and political environment in which they take place (Billig, 1988; Hopkins, Reicher & Levine, 1997; Sakki & Pettersson, 2016).

One way to analytically approach the argumentative character of (online) interactions is through the concept of *semantic barriers* (Gillespie, 2020). According to Alex Gillespie, semantic barriers are ‘meanings used to reinforce the semantic boundary between the self's views and the views of others, thus protecting the self's universe of meaning from being destabilized’ (Gillespie, 2020, p. 22). In intergroup interaction and confrontation, semantic barriers operate through three layers of defence: *avoiding*, where the perspective of the outgroup is disregarded; *delegitimising*, where the outgroup perspective is acknowledged but dismissed as invalid; and finally, *limiting*, where the outgroup perspective is not disregarded but its impact is limited. Avoiding can take place through denying certain groups of people (e.g., refugees or asylum seekers) voice in matters that concern them, or through *dehumanization* of the outgroup, constructing them as ‘less than human’ and thus lacking the right to a perspective (cf. Haslam & Loughnan, 2014). In terms of delegitimising, it can take the form of, for instance, framing the outgroup perspective as based on ignorance, incompetence or subjectivity, thus lacking a factual basis. Limiting, finally, is often constructed through disclaimers, ‘I see what you mean, but…’ followed by rationalizing and isolating arguments, such as when claims about discrimination are categorized as individual laziness or incapability. In sum, in line with Billig's (1987) theories of argumentation and counter‐argumentation, the core idea of semantic barriers is that when presenting our views, we do so in relation to existing or assumed alternatives – the views and positions of others – which serve as reference points for our own arguments and for how we position ourselves in the social world.

In the present study, we seek to add to the literature discussed above by analysing how a female right‐wing populist leader becomes positioned in online interactions in the ‘hybrid media system’ (Chadwick, 2017). Working within the field of communications studies, Andrew Chadwick (2017) coined the term ‘hybrid media system’ to refer to the co‐existence and mutual interactions in contemporary digitalised societies between different (more traditional and newer) media types. Within this system, according to Chadwick, actors ‘…can create, tap, or steer information flows in ways that suit their goals and in ways that modify, enable, or disable the agency of others, across and between a range of older and newer media settings.’ (Chadwick, 2017: xi).

We approach this topic through the perspective of critical discursive psychology (CDP, e.g., Edley, 2001; Wetherell, 1998), which through its consideration of the rhetorical and ideological constitution of discourse renders it particularly useful for studying online argumentation contestation and expressions of hate (Burke & Rowe, 2015; Cervone et al., 2021). In so doing, our aim is to contribute to the social and discursive psychological study of right‐wing populism theoretically, by showing the ways in which notions of gender intertwine with ‘classic’ right‐wing populist discourses in online interactions; methodologically, through the tools of CDP and the concept of semantic barriers, analysing how the same rhetorical resources can be used for opposing ends in such interactions; and empirically, by providing novel research on female populism.

## METHOD

The material for this study is comprised of two separate yet interlinked social media datasets. The first one is constituted of the comments (*N* = 334) associated with the magazine *Suomen Kuvalehti's* Facebook post on 8th October 2021 about the article on Purra. The second dataset comprises the follower replies (*N* = 176) to Purra's tweet on 9th October 2021 about the article and the Facebook comments it had triggered. The article constituted the first major news story about Purra following her election as president of the Finns Party. The Facebook and Twitter datasets analysed in the present study were explicitly linked to the article. Thus, these datasets were chosen as they together enable a discursive analysis of how Purra as a political leader is positioned and how female populism is constructed in the hybrid media system, that is, in the mutual interactions between different types of media (Chadwick, 2017). As we saw above, in her tweet Purra explicitly referred to comments containing derogating and dehumanizing hate‐speech against her. At the time of data collection (mid‐October 2021) some comments – including the one Purra referred to – had been deleted due to their harsh contents, a methodological limitation we discuss in the last section of this article. Both the Facebook account of *Suomen Kuvalehti* and Purra's Twitter accounts have approximately 34,000 followers, respectively.

The data were analysed in their original Finnish language. The English translations in the analysis below have been done by the authors. Being fluent in both Finnish and English, we have strived to capture the original meanings of the comments. Yet we recognize that some idiomatic expressions and slang words are hard to translate directly, and in such cases, we have included the original Finnish formulation and explained it as best we can. All extracts in their original Finnish form can be found in Appendix S1.

Conducting research on online data involves distinct methodological challenges (e.g., Crosset, Tanner & Campana, 2019) and ethical considerations (Ditchfield, 2021). Regarding the former, a limitation of this study is that due to the character of the discourse we studied – online discussions featuring a considerable amount of hateful and derogatory talk – some comments had been deleted by the page moderators before we could access them. This entails that some of the original meanings and argumentative aspects of the data may have been lost. In terms of ethical challenges, such as the preservation of commenters' anonymity, although we cannot know whether the followers posted their comments in their own names or under pseudonyms, we have pseudonymized the comments shown in the analysis below. However, as both social media threads are publicly available, it could be possible to trace the accounts of the commenters, an issue that we recognize as ethically troublesome. Furthermore, as some of the discourse we analyse contains derogatory and offensive speech, its reproduction here can be regarded as problematic, as it could serve to increase its societal influence. Nevertheless, we consider displaying the comments necessary for the sake of transparency of the discourse analytic process. Comments containing, for instance, mere curse‐words or insulting outbursts, have not been included in the analysis below.

Our analysis of these data relied on the methodological approach of CDP and its central analytic concepts *interpretative repertoires* and *subject positions*. The concept of interpretative repertoires was introduced by Gilbert and Mulkay, (1984) and brought into social psychological research by Potter and Wetherell, (1987) in their classic work on discursive psychology. Interpretative repertoires are sets of discursive resources that are intertwined with any community's common sense (Edley, 2001). They are deployed in people's talk as means to (re)construct shared social understandings and, due to their argumentative character, to construct alternative versions of reality and to serve particular social functions, depending on the discursive context (Wetherell, 1998).

According to Nigel Edley (2001), interpretative repertoires are typically (though not always) aligned with corresponding subject positions. From a discursive perspective, subject positions connect[s] the wider notions of discourses and interpretative repertoires to the social construction of particular selves' (Edley, 2001, p. 210). They are locations or identities that individuals can construct for themselves and others through particular ways of talking. In our analysis, then, we were interested in the ways in which, drawing on ideologically rooted and culturally shared knowledge, the commenters discursively constructed female populism (interpretative repertoires), how they in so doing in their talk constructed different identities or locations (subject positions) for Purra. In particular, our interest lay in how these interpretative repertoires and subject positions were constructed in argumentation and counter‐argumentation between the commenters to serve particular social and political functions.

Our analytic process involved three intertwined stages according to the procedure proposed by Sakki and Pettersson (2016). First, we systematically read and reread in order to analyse the consistency and variability (Potter & Wetherell, 1987) within the discursive patterns (*analysis of content*). We discussed these initial findings and as a result, arrived at the interpretation of four main discourses, or here, interpretative repertoires, in the two datasets, as detailed below. Second, following the central thrusts of CDP and rhetorical social psychology, we proceeded to analyse in detail how these repertoires were discursively and rhetorically constructed, that is, what culturally shared knowledge (Wetherell & Potter, 1992) and rhetorical strategies (Potter, 1996) they relied on (*analysis of form*). This stage also involved the analysis of how, within each of these repertoires, comments either defending or criticizing Purra constructed opposing subject positions for her through dialogue and argumentation. In conjunction, we examined the ways in which the commenters positioned themselves and each other through the construction of semantic barriers (Gillespie, 2020). Finally, we elaborated on the discursive functions of these discursive patterns, that is, what they are able to achieve in a social and political sense (*analysis of function*). In the following section, we demonstrate our findings.

## ANALYSIS

Our analysis of the Facebook and Twitter comments on the *Suomen Kuvalehti* article and Purra's tweet about it identified four distinct, yet to some extent overlapping interpretative repertoires about Purra and her politics. Within these repertoires, we explored the ways in which Purra was discursively positioned by the commenters through argumentation and counter‐argumentation. The comments that did not fall into any of the aforementioned repertoires and positions were either completely detached from Purra and the article, discussed these in neutral terms (e.g., Purra as an introverted person; technical difficulties with accessing the article), or centered on insulting other commenters without connection to Purra.

The four interpretative repertoires about female populism that we identified were those of (1) female populism as ‘(un)fit, (2) female populism as (non‐)racist, (3) female populism as (un)patriotic, and (4) female populism as a target or source of hate. According to our analyses, within these repertoires Purra was positioned and counter‐positioned as (1) either a ‘fit’ or ‘unfit’ politician, (2) a racist or non‐racist, (3) a patriot or non‐patriot, (4) a victim or culprit of hate‐speech or misogyny. As we will see, the ways in which the positions were discursively constructed varied somewhat between the two datasets, the Facebook comments focusing more on the politics of Purra and her party (the repertoires of (fit, racism and patriotism), whereas the repertoire of female populism as a target or source of hate was more common in the Twitter data. In order to capture the interconnection between the Facebook and Twitter data, we combine the two datasets in the presentation of our findings below.

### Repertoire 1: Female populism as (un)fit

#### Positions: Purra as fit versus unfit

The scrutinizing spotlight on female politicians, as compared to that of their male colleagues, is a well‐documented one within research on female political leaders (Carlin & Winfrey, 2009; Hall & Donaghue, 2012; Sakki & Martikainen, 2022; Sorrentino & Augoustinos, 2016). The first interpretative repertoire examined in the present study – that of *female populism as (un)fit –* afforded two competing subject positions that commenters constructed for Purra: those of a fit or unfit politician, as exemplified in the extracts below:

*Extracts 1–2 (Facebook):*
***Liisa:***
*Beautiful and gorgeous and clearly beats the government‐fiver in terms of intelligence*.
***Timo:***
*In terms of intelligence, she's lightyears behind e.g. Li Anderson (sic) and she doesn't even look that special*.

In the exchange above, we can see, first, that whether Purra is a fit politician is discursively evaluated based upon her looks and alleged intelligence, and second, how this positioning is imbued with gendered connotations. A classic juxtaposition is made between Purra and her political adversaries – in this case the five female ministers in the Finnish national government at the time – with intelligence attributed either to Purra (Extract 1) or to government representatives (Extract 2). In the present examples, an explicit contrast in terms of intelligence is constructed between Purra and the Finnish Minister of Education, Li Andersson of the leftist party. Purra's alleged inferiority in this regard is expressed metaphorically by the speaker in Extract 2 as her being ‘lightyears behind Andersson’, which, in Gillespie's (2020) terms, serves to undermine the other's (Purra's) competence, thus constructing her perspective as invalid. An interrelated point of judgement to that of her intelligence revolves around Purra's looks, the commenter in the first extract highlighting her beauty and the second one mitigating it: ‘and she doesn't even look that special’. These extracts illustrate the gendered connotations inherent in constructions of women politicians in public discourse: they tend to focus on their appearance and evaluate their beauty in conjunction with assumptions of their (lack of) intelligence (Carlin & Winfrey, 2009; Sakki & Martikainen, 2022), a topic that is seldom a concern in discussions about their male colleagues.

Within the interpretative repertoire of female populism as (un)fit, many comments discussed Purra's competence and politics in relation to the good it does or does not do for the Finnish people, as in Extracts 3 to 4 below:

*Extracts 3–4 (Twitter):*
***Kaisa:***
*Mauri, yes this Mrs Purra is surely gifted and even wise, but she has the same tendency as HA* [Jussi Halla‐aho], *accepting nasty and even ugly language and talks as a radical, thinking about only herself and her party. She might say that she's on Finland's side, but in the end she's the opposite*.
***Anne:***
*Kaisa, Purra may be smart, but wise she is not. They are two different things*. […] *Then we have emotional intelligence. That Purra lacks. She also lacks empathy, love for one's neighbour, tolerance, open‐mindedness, desire to help and many other good characteristics that a person, also a politician, should own. This woman knows how to incite to confrontation, hate and resentment and encourages this in her party. And then she pretends to be a mother‐person!*

In the exchange in Extracts 3–4 above, Purra's intelligence is not disputed; however, she is accused by both speakers of using her skills for her own good and for the ill of the Finnish people and nation. The first comment (Extract 3) begins with a disclaimer ‘yes, this Mrs Purra may be talented and even wise, but…’, whereafter she is aligned with her predecessor, Jussi Halla‐aho, as someone who despite claiming to act on behalf of the people, ends up doing the opposite. The explicitly gendered expression ‘Mrs Purra’ is interesting, as such formulations are considered old‐fashioned and are very seldom used in everyday talk in Finnish. In its position as part of the disclaimer, the formulation can therefore be read as a sarcastic, belittling one. The speaker in Extract 4 – replying directly to the comment in Extract 3 – denies the claim that Purra would be wise and instead asserts that Purra uses her skills to do harm. This argument is rhetorically strengthened through the strategy of *listing* the claims about Purra's character: ‘She also lacks empathy, love for one's neighbour, tolerance, open‐mindedness, desire to help and many other good characteristics…’ and when accounting for her wrongdoings: ‘confrontation, hate and incitement to resentment’. Finally, the speaker contrasts all this alleged evil to Purra's role as a mother, phrased as an accusation: ‘And then she pretends to be a mother‐person’! Together, these formulations illustrate gendered meanings oftentimes inherent in the commenters' descriptions of Purra's character, appearance and competence, and how these meanings could be mobilized to position her in a positive (as in Extract 1) or negative light (as in Extracts 2–4); as a fit or unfit politician.

### Repertoire 2: Female populism as (non‐)racist

#### Positions: Purra as racist versus non‐racist

A vast amount of research on far‐right and right‐wing populist discourse has been devoted to how speakers manage the issue of racism in their talks. This research has shown how racism can be downplayed through *discursive deracialisation* (Reeves, 1983; cf. e.g., Pettersson & Augoustinos, 2021), that is, by replacing notions of race and ethnicity with other constructs, such as those of the nation and national belonging, and through accusations, denials and reversals of racism (van Dijk, 1992, 1993, cf. Goodman & Johnson, 2014; Richardson & Colombo, 2014; Sakki & Pettersson, 2016). In the following extracts, we can see how the commenters manage the issue of racism in their talk, using it as a discursive resource both to defend Purra and to contest her politics:

Extracts 5–6 (Facebook):
***Aino:***
Even the best grades are not enough when one's worldview is regressive, racist and cliquish. Turned inwards. Doesn't resonate with the current global world and its increasing demands. The development has stopped.
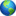

***Katja:***
Aino, it's good to pull the race card when nothing else works. It's strange that you're not allowed to defend the Finns. The need for help is closer than anyone wants to understand. The elderly, the young, families with children, single parents, they're right here, you don't have to look thousands of miles away. How can Finns provide for all newcomers, when taxpayers are not taken care of.

In the comments above, the first one explicitly positions Purra as racist and lacking perspective, whereas the second one instead implies that the position of Purra is one that leads to better care and support being provided for the Finnish people. Contrary to a common pattern of avoiding direct accusations of and references to racism in everyday talk (e.g., van Dijk, 1992), but in line with research on derogatory speech in the online context (e.g., Burke & Goodman, 2012; Cervone et al., 2021; Merrill & Åkerlund, 2018), the race talk in the extracts above is explicit and contains strong accusations of racism. A closer examination of the entries above allows us to see the extent to which they – despite producing the opposite discursive functions – rely on similar rhetorical strategies. Both comments deploy the rhetorical strategy of *listing*, describing Purra as ‘regressive, racist and cliquish’ in the first comment, and positioning her as standing up for ‘the elderly, the young, families with children, single parents’ in the second. Furthermore, both deploy what Wetherell and Potter (1992) called ‘liberal‐practical’ or self‐sufficient rhetorical arguments that are hard to refute, illustrated in the expression that Purra's worldview ‘doesn't resonate with the current global world (…) The development has stopped’ (Extract 5) and ‘It's strange that you're not allowed to defend the Finns’ (Extract 6). It is interesting to note how, in these extracts, the commenters construct ‘racism’ through invoking shared, place‐specific meanings and understandings of the term. In Extract 5, the speaker accuses ‘racists’ of looking ‘inward’, not abiding by ‘global demands’, whereas in Extract 6, those who want to address racism as a societal problem in Finland are allegedly ‘looking a thousand miles away’. Indeed, in both extracts, we can see how the speakers construct racism as a ‘global’ concern that carries limited or no relevance in the Finnish context, and where those ‘in need’, ‘the elderly, the young, families with children, single parents’, are categorized as belonging to the (white) Finnish majority.

In terms of argumentation and counterargumentation, as a response to the first comment that constructs Purra as racist, the second one undermines the importance and legitimacy of this construction, first, through positioning Purra as a defender of the Finnish people against refugees and asylum‐seekers who allegedly parasite on the Finnish welfare and tax‐money, and second, through the accusation of an (illegitimate) accusation of racism*:* ‘It's good to pull the race card…’ (Augoustinos & Every, 2010; Durrheim, Greener & Whitehead, 2015; Goodman & Burke, 2010; van Dijk, 1992; Whitehead, 2009). The speaker in Extract 5 delegitimizes the other's perspective, not through mitigating the other's intelligence as in Extract 2 above, but through accusing the other (implicitly, Purra) of racism (cf. Gillespie, 2020). In Extract 6, in turn, the formulation ‘pull the race card when nothing else works’ serves to construct this accusation as desperate, lacking grounds and thus illegitimate. In sum, the extract above illustrates how racism can be constructed through argumentation and dialogue to serve opposing discursive functions, that is, for portraying the speaker's own arguments as non‐racist and the other's as racist (cf. Durrheim et al., 2015).

### Repertoire 3: Female populism as (un)patriotic

#### Positions: Purra as a patriot versus non‐patriot

As we will see in the following extracts, the discursive constructions and accusations of racism strongly intertwined with the third interpretative repertoire examined here: that of *female populism as (un)patriotic*. Previous research on populist radical‐right discourse in the Finnish context (e.g., Sakki & Pettersson, 2016; Sakki & Pettersson, 2017) has shown that the collective memory of the wars against Russia during WW2 is a frequently deployed rhetorical resource in the construction of the self as acting in the interest of the nation and the people, and of the other as betraying these very ones. An example of such discourse is provided in the arguments and counterarguments presented in Extracts 7 to 8 below:

*Extracts 7–8 (Facebook):*
***Mirja:***
*My father was a war veteran. He never accepted that racism and discrimination were justified through patriotism and didn't go to war to defend such ideals. I'll end the discussion here, since I'd rather use my time for exchanging constructive thoughts*.
***Katja:***
*Mirja, I don't believe your stories. No war‐veteran fought for independence for it to be broken by people like you. You throw in the race‐card again, although it's not about racism. It's realism about what is and isn't possible. You truly are the worst kind of racist*.

In the exchange above, the commenters are arguing about the meanings of racism versus non‐racism, and patriotism versus non‐patriotism. Similar to what we saw in Extract 5 above, the speaker in the first comment criticizes those who abuse the notion of patriotism for racist or discriminatory ends, whereas the second commenter accuses the first one of ‘playing the race card’ (cf. Burke & Goodman, 2012; Durrheim et al., 2015; Goodman & Burke, 2010), which again functions to undermine the importance and legitimacy of the point about racism made by the previous commenter, and thereafter constructs an explicit reversal of racism (van Dijk, 1992) formulated in the extreme (Pomerantz, 1986): ‘you truly are the worst kind of racist’. Even though Purra is not directly mentioned in this comment (nor in the ones in Extracts 8–10), all the comments studied here are part of the threads connected to the article about Purra (the Facebook data) or replies to her tweet (the Twitter data), we interpret them as nevertheless being related to Purra, the Finns Party and/or the ideology that they promote. In this regard, they can be interpreted as constructing particular versions of (Purr'as) female populism.

In the first comment, thus, the ‘patriotic’ agenda of the Finns Party is indirectly accused of being racist through the category entitlement (Potter, 1996) of the speaker's war‐veteran father who ‘never accepted that racism and discrimination were justified through patriotism’. As a direct counter‐argumentation to this statement, the reply‐comment invokes ‘all war‐veterans’, arguing that none of them defended the country for it to be destroyed by the likes of the first speaker. In this exchange, in sum, we can see how accusations and reversals of racism on one hand, and the construction of the category of war‐veterans as inherently patriotic, good and non‐racist on the other, are used as rhetorical resources for either defending or criticizing the politics of Purra's party, and for constructing this as either patriotic or unpatriotic. In the exchange above, we can in Gillespie's (2020) terms see an example of the *avoiding* layer of semantic barriers: by mobilizing the sanctity of the war‐veterans as a rhetorical argument, the speakers position themselves as patriots and unequivocally dismiss the other's perspective as unpatriotic, racist and worthy of no attention, as the last sentences (‘I'll end the discussion here, since I'd rather use my time for exchanging constructive thoughts’ and ‘You truly are the worst kind of racist’) in each comment fortify.

In the following extracts, the speakers argue between the meanings of patriotism versus non‐patriotism through accusations of communism and Russian‐mindedness, a common rhetorical trope in Finnish populist radical right and anti‐immigration discourse (cf. Sakki & Pettersson, 2016; Sakki et al., 2017). Indeed, the speaker in Extract 9 below replies to Purra's tweet and attributes blame for the hatred against her to the ‘leftists and greens’:

*Extract 9 (Twitter):*
***Hannu:***
*Correct – the #leftist greens have returned to the red Russians' [punaryssien] rhetoric of 1918 #hag‐government [akkahallitus]*

The speaker in Extract 9 accuses leftists and greens of deploying the ‘rhetoric of the reds’, a reference to the 1918 Finnish civil war, where the two opposing sides were called the ‘whites’ and the ‘reds’, the latter having connections to communist Russia. Previous research examining Finnish far‐right discourse from the early 20th century until today has demonstrated that the strong presence of anti‐communist rhetoric of the past translates into anti‐immigration discourse in the present (cf. Sakki et al., 2017; Silvennoinen, Tilkka & Roselius, 2016). Yet, the use of the communist‐label as a derogatory accusation of lack of patriotism and even treason to the nation lingers on in Finnish far‐right and anti‐immigration political discourse, and the association between liberal, leftist, and green politicians and communism is a common rhetorical manoeuvre in such talk (Sakki & Pettersson, 2016).

The gendered character of the discourse around patriotism is articulated through the hashtag *#akkahallitus* ‘hag‐government’ at the end of the comment in Extract 9, which produces a direct link between communism and the female‐dominated Finnish government. This type of communication – using the affordances of social media, such as hashtags, to present strong accusations without expressing explicit personal stances – has been called ‘digital voicing’ in previous research (Pettersson & Sakki, 2017). Here, it allows the speaker to add a gendered connotation to the ‘red’ communist label, associating it with Purra's political antagonists and women in power in Finnish politics. Yet, the implicitness enabled by the use of a hashtag – rather than explicit statements – allows the speaker to dodge potential accusations of misogyny.

Whereas the discursive association between leftist and green politicians and communism is a well‐documented one in previous research on political discourse, far less common is the opposite, that is, that the populist radical‐right are the ones to be annotated such labels. An illustration of such a construction given in Extract 10 below:

*Extract 10 (Facebook):*
***Jukka:***
*Liisa, how to defend Finland and the Finns when one is so tilted towards Russia, like the communists once‐upon‐a‐time. Against both the native Finns and the EU, like Soini [former president of the Finns Party], who wanted to form his own Finns Party‐land [persulandian] with Russia*.

In contrast to what we saw in the Twitter comment in Extract 9, in the comment above (which was part of a comment thread emanating from Liisa's comment in Extract 1) an explicit link is drawn between the Finns Party on one hand, and Russia and communism, on the other. These accusations draw upon the collective memories of Finland's wars against Russia and on the subsequent complex relationship Finland has to its big Eastern neighbour. Thus, constructing Purra's party as allied with Russia serves to accuse them of neglecting the Finnish people and being essentially unpatriotic. As the comments in extracts 9 to 10 show, the same rhetorical strategy – accusations of communism or allegiance with Russia – was used to construct Purra's party either as an unpatriotic national traitor (Extract 10), or conversely, as a counterforce to this very traitor, the leftist‐green female government (Extract 9). In both cases, the accusation of communism serves, similar to what we saw in Extracts 6–7 above, to create semantic barriers between the speakers, allowing them to avoid taking any consideration of the other's perspective in their talk.

### Repertoire 4: Female populism as a target or source of hate and misogyny

#### Positions: Purra as a victim or culprit of hate and misogyny

The last interpretative repertoire we identified through our analysis constructed female populism as either a target or source of hate‐speech and misogyny. We made interpretations of this repertoire mainly in the Twitter data, where many comments – including the ones shown in Extracts 11–13 below explicitly related to Purra's tweet about the hatred against her that the article had evoked. Within this repertoire, the argumentation revolved around issues of guilt and victimhood, positioning Purra (and, by implication, the Finns Party) either as guilty or victims of hate‐speech and misogyny. Following Boromisza‐Habashi (2012), our analysis in this section focuses specifically on how the speakers construct and attach meanings to the notions of hate‐speech and misogyny, and not on seeking examples, according to legal or academic definitions, of their occurrence in the talk. In many cases, the commenters discussed Purra's part in ideologically or politically and ethnically or culturally motivated derogatory speech, as displayed in Extract 11 that shows a direct reply to Purra's original tweet:

*Extract 11 (Twitter):*
***Markus:***
*This is the allowed kind of hate‐speech in Finland. If those comments would have been directed e.g., at migrants, we would have seen judgements. Funny double‐standards*.

In the extract above, the speaker aligns with Purra's statement (her original tweet), comparing the hate‐speech against Purra to that directed at migrants, and ironically arrives at the conclusion that the sanctioning of the former and judging of the latter represents a ‘funny double‐standard’ in Finnish society. Through this discourse, Purra is positioned as a victim and target of hate‐speech, suffering unduly as society does nothing to stop it.

In comments opposing Purra's claim, the argument about double‐standards in Extract 11 above is here used for the opposite end: to position Purra and her party as guilty and ‘non‐Finns’ as the victims, as the following replies to Purra's tweet illustrate:

*Extracts 12‐13 (Twitter):*

***Jussi:***

*You* [Purra] *just gave Tynkkynen a new plume in his hat* [sulka hattuun] *by accepting his insults towards Muslims. Is it so that you can't stand other's statements, but you are the victims, if you cannot slander others? Pretty egocentric approach!*

***Janne:***

*You* [Purra] *could think about who brought the asshole [persläpi] and foreign species vocabulary into parliament. Perhaps someone in your own ranks?*

In Extract 11 above, the speaker draws upon the case of the Finns Party politician Sebastian Tynkkynen, who has been prosecuted for criminal hate‐speech against Muslims on three occasions, but who continues to pursue a successful political career in the Finns Party as MP and vice‐president (cf. Pettersson, 2019). Here, the speaker depicts Purra, directly addressed in the word ‘you’ in the comment above, as sanctioning hate‐speech on behalf of her own party but condemning that of others, thus positioning her as hypocritical and egocentric. The positioning of Purra as guilty of inciting to hate‐speech becomes more forceful in the following extract (13), where Purra and the Finns Party are explicitly accused of having brought derogatory, dehumanizing concepts into the political and societal debate. Indeed, in a similar vein to what we saw in the discursive accusations, denials and reversals of racism discussed under the interpretative repertoire of racism above, corresponding discursive manoeuvres can be done in discursive constructions of hate‐speech to construct guilt or victimhood.

In the analysed data, intertwined with the interpretative repertoire female populism as a source of hate‐speech on political‐ideological or ethnic‐cultural grounds was the one constructing female populism as a source specifically of misogyny. As in the comments analysed in Extracts 11 to 13 above, also here the commenters argued about the meanings attached to guilt versus victimhood, constructing corresponding positions and counter‐positions for Purra:

*Extracts 14‐15 (Twitter):*
***Sofia:***
*If one reads stories on any female politician, they're always like that. Especially those in power. At worst they're tough violent fantasies. So this can't really have been a surprise? This is what ”discussion” means today, one can say anything*.
***Raija:***
*This is misogyny. Men who fear women cannot express their fears with other words – unfortunately*.

In the extracts above, which again are direct replies to Purra's tweet, the derogatory comments against her are discursively constructed as examples of misogyny. Both speakers deploy the rhetorical strategy of consensus warranting (Potter, 1996) when accounting for misogyny as something inevitably facing female politicians in contemporary society. In these comments, women in general, and Purra as a woman by implication, are positioned a targets of misogyny. Even so, these constructions do not communicate clear empathy, pity, or support for Purra, but rather, serve to position her in the same category as any female politicians, or indeed women in general, who must come to terms with the societal problem of hatred against women. By contrast, in the final two replies to Purra's tweets shown below Purra and her supporters are accused of being the main culprits of producing and spreading misogyny in society:

*Extracts 16–17 (Twitter):*
***Erkki:***
*Sure, that's sad reading, but have you Riikka checked what your supporters continuously churn out about Marin and the other government ministers. This kind of communication mostly comes from those who support Riikka. Could you do something yourself?*
***Jakob:***
*The Finns Party churn out muuuch worse comments whenever there's an article about Marin. Marin doesn't care and doesn't complain in social media – that's indeed why she is a respectable professional, an action‐oriented politician*.

In both comments above, the speakers make explicit comparisons between the hatred directed against Purra and that facing the Finnish PM Sanna Marin and other female ministers. Extract 16 starts with a disclaimer ‘Sure, that is sad reading, but…’ and continues to confront Purra with the hatred that her supporters direct against Marin and her colleagues. One may interpret this argumentation as creating the limiting layer of semantic barriers (Gillespie, 2020): the disclaimer in connection with the alleged dichotomy between the (limited) hatred directed at Purra and the (excessive) hatred other women politicians face serves to mitigate the significance of Purra's perspective. Interestingly, furthermore, the speaker uses the first name ‘Riikka’ when referring to Purra, and Marin's surname when talking about the PM. This emphasizes the contrast constructed between the two, as the use of first name serves to belittle the object of talk, and the use of surname to indicate respect and dignity (Carlin & Winfrey, 2009; Sensales, Areni & Dal Secco, 2016). In the final sentence formulated as a question, the speaker explicitly urges Purra to take action against the hate‐speech against Marin and her government. In Extract 17, a comparison is again made between Purra and Marin, constructing the latter as a target of much more severe hatred, yet as a brave and strong politician. Through this construction, Purra, by contrast, is positioned not only as a culprit inciting hate‐speech through her followers, but also implicitly as weak and complaining. In sum, as Extracts 14 to 17 demonstrate, the same rhetorical manoeuvre – the construction of a common category of female politicians as targets of hate‐speech – could be used to construct repertoires of female populism as the source or target of hate‐speech, and, correspondingly, to position Purra either as a target (Extracts 14–15) or as inciting to hate‐speech and misogyny (Extracts 16–17).

## DISCUSSION

This study has explored the discursive construction in the hybrid media system (Chadwick, 2017) of interpretative repertoires of female populism and corresponding subject positions for a female president of a populist radical right‐wing party. Apart from shedding empirical light on the role of women in populist movements, the study sought to make a twofold contribution to the literature on populist and radical right discourses online: to explore the ways in which notions of gender are mobilized in such discourses and examine their argumentative character. In terms of the first aim, we have seen how gender was intimately associated with the discursive constructions of Purra's politics as (un)fit, connecting her alleged (un)intelligence with remarks about her appearance and with comparisons to her political adversaries, the female ministers in the Finnish national government. These comments resonate with previous research highlighting the scrutinization of female politicians as compared to their male colleagues: the evaluation of the competence of female politicians is inherently intertwined with discourse assessing their beauty and the degree of their intelligence (e.g., Carlin & Winfrey, 2009; Hall & Donaghue, 2012; Sakki & Martikainen, 2022).

The interpretative repertoire of female populism as (un)patriotic was characterized by discursive accusations of communism and connections between communism and womanhood. This finding is in line with previous research on the construction of otherness in populist radical right discourse, where a key figure in such discourse is that of the female, left‐leaning feminist politician (e.g., Keskinen, 2013; Sakki & Pettersson, 2016). Within the interpretative repertoire of female populism as a target or source of hate‐speech and misogyny, the explicitly gendered talk constructed misogyny both as something that faces female politicians – including Purra – across the political spectrum, and as the fault of Purra and her followers, who were constructed as accountable for the greatest extent and most severe forms of hatred and misogyny. The fact that this interpretative repertoire was discernible mainly in the Twitter data can be regarded as a ‘natural’ consequence of Purra's original tweet. Nevertheless, it may also hint towards a somewhat different discursive climate on Facebook as compared to Twitter, the latter being characterized by emotional and forceful language (cf. Törnberg et al., 2021).

Turning to our second aim, our analysis showed that the subject positions and corresponding counter‐positions associated with the interpretative repertoires described above of Purra as a *fit* versus *unfit politician*, *racist* versus *non‐racist, patriot* versus *non‐patriot* and *victim or culprit of hatred and misogyny* were constructed in argumentation and dialogue between the commenters of the Facebook and Twitter posts. Indeed, oftentimes the exact same arguments and rhetorical strategies – attributions or denials of intelligence and beauty, accusations of racism or communism, and of guilt or victimhood with regards to hate‐speech based on ideology, ethnicity or gender – were exploited for the opposite ends, that is, for positioning Purra and her politics in either favourable or unfavourable light. This resonates with Billig's (1985, 1987, 1988; Billig et al., 1988) work on the argumentative and dilemmatic character of human reasoning, thinking and (social) meaning making.

As our analyses further demonstrated, not only did the speakers construct competing subject positions for Purra through argumentation and counter‐argumentation, but in so doing, simultaneously created semantic barriers between self and other through avoiding, delegitimizing or limiting the other's perspective (cf. Gillespie, 2020). Previous research has indeed demonstrated that argumentation between opposing ideological positions and political identities in the social media environment can increase polarization at a societal level (Törnberg et al., 2021). Our study has explored how this polarization may take place at the micro‐level of interaction and showed how the struggle for political meanings is deeply intertwined with the (re)production of intergroup hostility and polarization in the online sphere.

Our study has demonstrated that both the comments that supported and those that criticized Purra expressed strong accusations and denials of racism, alongside accusations of *accusations of* racism, which constructed the other and their comments as inherently bad and worthy of no acknowledgement or respect (cf. Gillespie, 2020). Correspondingly, accusations of communism and pro‐Russian sentiment was not only directed at Purra's political (female) antagonists, as documented in previous studies (e.g., Sakki & Pettersson, 2016) but surprisingly, also at Purra and her party. We interpret this finding as revealing something about the cultural and historical significance of the notion of communism in the Finnish context: it evokes memories of Finland's traumatic civil war and wars against Russia and is a rhetorically powerful tool for constructing the other as unpatriotic – even evil. In other words, the ‘communist card’ seems as forceful as the ‘race card’ (Durrheim et al., 2015) in the context of Finland.

This supports the argument that in order to capture its ideological features, political discourse must always be examined as part and parcel of its surrounding historical and political context, which the critical discursive psychological approach allowed us to do. Indeed, the approach is particularly well suited for studying online (political) communication as it allows, on one hand, to study this discourse at the fine‐grained micro‐level of argumentation, yet on the other, to consider the broader ideological patterns of meaning that it is imbued with (Burke & Rowe, 2015; Cervone et al., 2021; Pettersson & Sakki, 2017, 2020). Indeed, we hope that our study has contributed to social psychological research on right‐wing populism, political polarization, and social constructions of racism, hate‐speech and misogyny, first, by investigating such constructions in the interactional context of the hybrid media system (Chadwick, 2017), where locally situated meanings, media discourse and political rhetoric intertwine; and second, by showing the utility of a critical discursive psychological perspective that integrates considerations of the micro‐ (i.e., the deployment of rhetorical and discursive manoeuvres) and macro‐ (i.e., the construction of socially and culturally situated meanings) dimensions of online discourse on political issues. Further deepening our understanding of how contestation and polarization about societally pressing topics takes place in online, grassroot level interactions, remains a pressing topic for social psychological research.

## AUTHOR CONTRIBUTIONS

**Katarina Pettersson:** Conceptualization; data curation; formal analysis; methodology; writing – original draft; writing – review and editing. **Inari Sakki:** Conceptualization; formal analysis; funding acquisition; methodology; project administration; writing – review and editing.

## CONFLICT OF INTEREST

All authors declare no conflict of interest.

## Data Availability

The data used in this study are publicly available through the following links: https://twitter.com/ir_rkp/status/1446686567821611009; https://www.facebook.com/SuomenKuvalehti/posts/10159920906412847.
