# Supplementary material for: ‘You truly are the worst kind of racist!’: Argumentation and polarization in online discussions around gender and radical‐right populism
Source: Br J Soc Psychol. 2022 Jun 29;62(1):119–35. doi: 10.1111/bjso.12557 (PMC10083942; doi:10.1111/bjso.12557)
Supplement: Supplementary file 1 [file BJSO-62-119-s001.docx]

**Appendix A: Extracts in Finnish**

Purra’s Tweet:

”Kylmät väreet käyvät inhosta tämän naisen avatessa suunsa, inhon väreet, jota tunnetaan verenimijöitä kuten paarmat, punkit, kohtaan.”

Tällaisia kommentteja löytyy paljon SK:n fb-sivulla minusta kertovan juttulinkin alla.

Käy lukemassa itse juttu. Linkki 👇

*Extract 1 (Facebook):*

*Kaunis ja upea ja älykkyydeltään mennen tullen päihittää hallitus viisikonkin.*

*Extract 2 (Facebook):*

*Älykkyydeltään on esim.Li Andersonia valovuosia jäljessä eikä nyt niin erikoisen näköinenkään ole.*

*Extract 3 (Twitter):*

*XX niin,tämä rva Purra on varmasti lahjakas ja jopa viisaskin,mutta hänen taipumuksensa on samoin kuin H.A ;lla ,hyväksyy ikävät ja jopa ruman kielen käytön,sekä on puheissaan radikaali,vain omaa ja puoluettaan ajatteleva henkilö.Vaikka hän sanoo olevansa Suomen asialla,mutta loppupeleissä se menee päinvastoin.*

*Extract 4 (Twitter):*

*XX Purra voi olla älykäs, mutta viisas hän ei ole. Nehän ovat kaksi eri asiaa Älykäs voi selvitä tilanteesta, johon viisas ei olisi joutunut.*

*Sitten on vielä tunneäly. Sehän Purralta puuttuu. Puuttuu myös empatiakyky, lähimmäisenrakkaus, suvaitsevaisuus, avarakatseisuus, auttamisenhalu sekä monta muuta semmoista hyvää luonteenominaisuutta, jotka ihmisestä, myös poliitikosta pitäisi löytyä.*

*Vastakkainasettelun, vihan ja kaunan lietsonnan tämä nainen osaa ja sitä suosii myös puolueessaan. Vielä olevinaan äiti-ihminen!*

*Extract 5 (Facebook):*

*Parhaimmatkaan arvosanat eivät riitä kun maailmankatsomus on taantumuksellinen, rasistinen ja nurkkakuntainen. Sisäänpäin kääntynyt. Ei vastaa nykyistä globaalia maailmaa eikä sen yhä lisääntyviä vaatimuksia. Kehitys on pysähtynyt. 
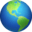
*

*Extract 6 (Facebook):*

*XX toi rasistikortti on hyvä vetää, kun ei muuhun pysty. Se on ihmeellistä, miksi ei saa olla Suomalaisten puolella. Hätää ja apuja on tarve lähempänä kun haluaa kukaan ymmärtää. Vanhukset, nuoret, lapsiperheet, yksinhuoltajat, ihan täällä ei tarvii hakea tuhansien km päästä. Millä kaikki tulijat pystyy Suomalaiset verovaroillaan elättämään, kun ei pidetä veronmaksajista huolta.*

*Extract 7 (Facebook):*

*Isäni oli sotaveteraani. Hän ei koskaan hyväksynyt sitä että rasismia ja syrjintää perustellaan isänmaallisuudella, eikä lähtenyt sotaan sellaisia aatteita puolustamaan. Jätän keskustelun tähän, koska käytän aikani mielummin rakentavien ajatusten vaihtamiseen.*

*Extract 8 (Facebook):*

*XX En usko sinun juttujasi. Kukaan sotaveteraani ei taistellut itsenäistä maata että se rikotaan, sinunlaistesi toimesta. Laitat rasistikortin taas, vaikka rasismista ei ole kyse. Realismia mikä on mahdollista mikä ei. Todellakin sinä olet rasisti pahemmasta päästä.*

*Extract 9 (Twitter):*

*Pitää paikkansa - #punavihervasemmisto on palannut punaryssien eli punikkien retoriikkaan vuodelta 1918 #akkahallitus*

*Extract 10 (Facebook):*

[*XX*](https://www.facebook.com/marjatee.18?__cft__%5b0%5d=AZWSESdF83S_X1fjBnicPmygChM1gniGwf9e6-BLR9uueydmwV4fwxOtfohD4Li4_UhirbEXIlc6SmUjGnMcO089JNh1u6Ji8Rn9JQsd3YcCYVeV3jnq8SXdW7drDQ_qqYLES4KfqOjKZ6Uv8sUM22Xv&__tn__=R%5d-R)*Miten puolustaa suomea ja suomalaisia, kun on niin kallelaan venäjään päin, kuten kommunistit aikoinaan. Sekä kantasuomalaisia ja EU:ta vastaan, kuten oli Soini, joka halusi luoda oman persulandian venäjän kanssa.*

*Extract 11 (Twitter):*

*Tämä on sitä suomessa sallittua vihapuhetta. Jos nuo kommentit kohdistuvat vaikka siirtolaisiin, sitä olisi jo tuomioita jaeltu. Hassu kaksoisstandardi.*

*Extract 12 (Twitter):*

*Juuri annoit Tynkkyselle uuden sulan hattuun hyväksymällä hänen solvaukset muslimeja kohtaan, että onks tää nyt niin, ettette itse siedä muiden sanomisia, mutta uhriudutte, kun ette saa avoimesti panetella muita? Aika egosentrinen lähestymistapa!*

*Extract 13 (Twitter):*

*Kannattaisi miettiä ketkä toivat Eduskuntaan persläpi ja vieraslaji nimittelyt. Löytyykö omista riveistä?*

*Extract 14 (Twitter):*

*Kun käy lukemassa juttuja kenestä tahansa nais-poliitikosta, jutut ovat aina tuollaisia. Erityisesti korkeassa asemassa olevista Pahimmillaan jutut on rankkaa väkivaltafantasiaa. Joten ei tämä varsinaisesti voinut yllätys olla? Tällaista "keskustelu" nykyään on, kaikkea saa sanoa*

*Extract 15 (Twitter):*

*Tämä on sitä naisvihaa. Miehet, jotka pelkää naisia, eivät osaa muilla sanoilla ilmaista pelkotilojaan - valitettavasti.*

*Extract 16 (Twitter):*

*Onhan tuo ikävää tekstiä, mutta oletko Riikka katsonut mitä kannattajasi jatkuvasti suoltavat Marinista ja muista hallituksen ministereistä. Tämänkaltainen viestintä tulee enimmäkseen Riikkaa tukevien joukosta. Voisitko tehdä itse jotakin?*

*Extract 17 (Twitter):*

*Paaaljon pahempia kommentteja ovat persut suoltamassa aina, kun kirjoitetaan artikkeleita Marinista. Marin ei niistä välitä, eikä valittele pahemmin asiasta somessa - siksi hän onkin kunnioitettavan ammattimainen, tekoihin keskittyvä poliitikko.*
